# Probiotics and prebiotics as alternatives to antibiotics in aquaculture: a systematic and bibliometric review of antimicrobial and antioxidant mechanisms

**DOI:** 10.3389/fmicb.2026.1784036

**Published:** 2026-04-01

**Authors:** Anjana Kalla Veedu, John Thomas

**Affiliations:** Centre for Nanobiotechnology, Vellore Institute of Technology (VIT), Vellore, India

**Keywords:** antimicrobial resistance, antioxidant, bibliometric analysis probiotics, bioactive compounds, prebiotics

## Abstract

**Background:**

The aquaculture industry has prominent role in the commercial production of fish protein needs of the world. Various technologies have emerged to improve the aquaculture industry. The alternative strategies for antibiotics and chemicals used in aquaculture to treat infectious disease are the potential probiotics and plant-derived prebiotics. As a result, farmers who depend on aquaculture were encouraged to use alternative strategies to prevent diseases. This review focuses on the effect of the antimicrobial and antioxidant properties of the prebiotic and probiotic bacterial strains on the aquatic pathogens.

**Methods:**

The systematic search related to probiotics and prebiotic effect on the aquaculture industry from Scopus databases was retrieved to identify the years of publications, countries from which it was published from 2015 to 2025. The bibliometric analysis performed provided comprehensive and detailed scientific data using quantitative tools like VOSviewer software.

**Results:**

A total of 339 publications and book chapters from the year 2005 to 2025 were retrieved for a major part of bibliometric analysis. A total of 1,475 research articles, reviews and book chapters were extracted for the VOS viewer networking. The countries from which maximum more research articles are India, China, and United States having 67, 50, and 36 publications, respectively. The top current keywords used are “probiotics,” “prebiotics,” and “aquaculture.”

**Conclusion:**

Both probiotic and prebiotics bacteria have a positive effect on immune responses, disease resistance, growth performance and tolerance against abiotic stressors of aquatic species is also discussed. The bioactive compounds and antioxidant effect of both probiotic and prebiotic bacteria are reported to have beneficial effects as a feed additive which can improve feed quality and animal health.

## Graphical Abstract

**Figure:**
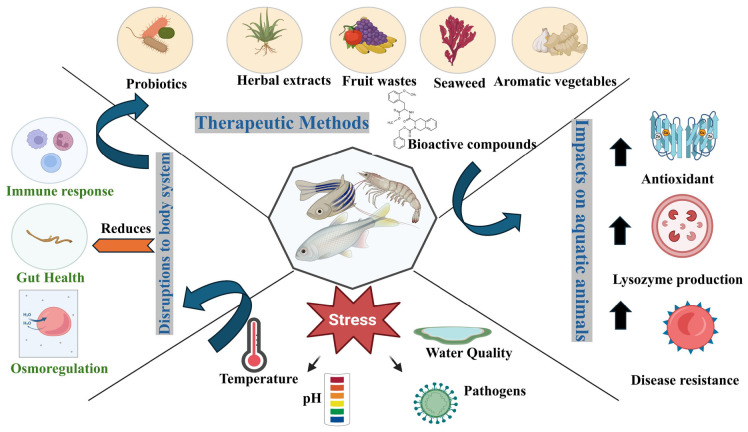
Therapeutic efficacy of probiotic and prebiotic against aquatic stresses.

## Introduction

The world’s rapidly growing food sector, with highly scalable opportunity, is known to be aquaculture industry. It has a prominent role in commercial food production because more than half the aquatic foods for human consumption are farmed. Inland aquaculture is known as aquatic farming which includes aquatic organisms like fishes, mollusks and planktons in the freshwater habitats like ponds, streams and other man-made environments according to the Food and Agricultural Organization data in 2025. For the sustainable growth and existence of agriculture and aquaculture their efficient management is important to secure a future supply of aquatic foods and agricultural products. The genetic diversity of aquatic species mainly the variety of color, shapes and many other characteristics of aquatic animals that we exploit shows the adaptability and resilience of species to changing environments. In the aquaculture industry, the economic loss was mostly caused by bacterial, fungal and viral diseases and was estimated at 6 billion USD in each year (Yue and Shen, 2022). The largest aquatic animal producing country is Asia while top aquaculture species producing country is China (Rohani et al., 2022). The effect of the overuse of antibiotics increases the number of antibiotic-resistant bacteria within the pathogenic bacteria in aquaculture farms and their entry into the food chain (Kari et al., 2022; Tanwar et al., 2014). In addition to their use, to control and treat bacterial diseases in both juvenile and adult fish, antibiotics also serve as prophylactic function in the developmental stage when it is incorporated into feed formulations. The current scenario of antibiotic resistance in aquaculture is the widespread resistance of aquacultural-based bacterial species, and the wide range of antibiotic classes were the evolvement of multi-drug-resistant organisms (Mohammed et al., 2025b). Aquaculture is practiced in the aquatic ecosystem and has an important impact on global food supply (Hu et al., 2025). The aquaculture field faces many issues. The major issue is strategic management of nutrient loaded effluents (Yang et al., 2025). In Indian aquaculture sector there are mainly three species of Indian major carps (IMC), *Cirrhinus mrigala* (Mrigal), *Catla catla* (Catla), *Labeo rohita* (Rohu), and exotic species, *Ctenopharyngodon Idella* (Grass carp), *Cyprinus carpio* (Common carp), and *Hypophthalmichthys molitrix* (Silver carp) (Suresh and Pillai, 2024). Gastrointestinal tract of humans is highly enriched by the diversified forms of microorganisms and their interactions with metabolites and structural compounds released by them (Berg et al., 2020). In recent studies, it has been reported that the gut microbiome has higher number of bacteria that produce short chain fatty acid (SCFA) (Ruan et al., 2020; Shanahan et al., 2021; Van Hul et al., 2024). Probiotics not only serve a crucial role in human gut health but also can maintains the water quality in aquaculture ecosystem.

Antibiotics are used as growth promoters which reduce infections and enhance fish health during their developing stages (Ringø, 2020). The declined growth of pathogenic bacteria leads to reduced productivity and increased market weight and hence the uncontrolled application of therapeutic agents has increased globally (Ringø, 2020; Concha et al., 2019). This uncontrolled usage of antibiotics is a risk factor to the organisms, the end consumer and the environment. Antibiotics can kill beneficial microorganisms which can lead to severe disturbance in the microbiota environment. Their use accelerates the resistance to bacteria and transfer of zoonotic resistant genes to human microbiota (Pereira et al., 2022). Furthermore, stress and environmental instability downregulate fish development by suppressing immune functions (Pereira et al., 2022). The mechanism behind the physiological characteristics of aquatic fishes, mainly immune parameters, is less studied (Reverter et al., 2021). Studies are focused on replacing the traditional fish feed with other protein sources (Amer et al., 2021). It is found that plant proteins can act as an alternative which is easily available and is cost effective (Kabir, 2021). It has been reported that antioxidants from natural sources that are ingested naturally by humans are less toxic and safer when compared to synthetics (Toner, 2004). Therefore, application of natural antioxidants in aquaculture feed enhances the growth of aquaculture organism (Hu et al., 2025).

In the aquaculture sector various drugs and chemicals were used to prevent and treat diseases (Dawood, 2021). However, several countries have restricted the use of these substances as they can harm human and animal health (Adel and Dawood, 2021). To overcome this problem probiotics and plant-derived proteins have replaced the chemicals used in aquaculture (Kari et al., 2022). Many studies have reported that agricultural waste has less effect on fish. Agricultural waste in feed of the fish can affect the seafood texture and its color (Sukri et al., 2022). Probiotics defend microbials by producing antimicrobial metabolites, which activate immune response to the pathogens (Pardo-Esté et al., 2024). The review aims to focus on the advanced and systemic analysis of mechanism of action of both probiotic and prebiotics in the fish growth and immunity focusing on their antibacterial, antioxidant and metabolic role.

## Materials and methods

(1). The review investigation was based on searching with keywords, including “aquaculture,” probiotics,” “prebiotics,” and “fish” from the Scopus databases.
(2). Review of effects of probiotics and prebiotics on aquatic animals.
(3). Review of antibacterial compounds in the probiotics and prebiotics which interact with the bacterial infections in the aquatic organisms.
(4). The different forms of probiotics.

### Data collection and retrieval methods

**FIGURE 1:**
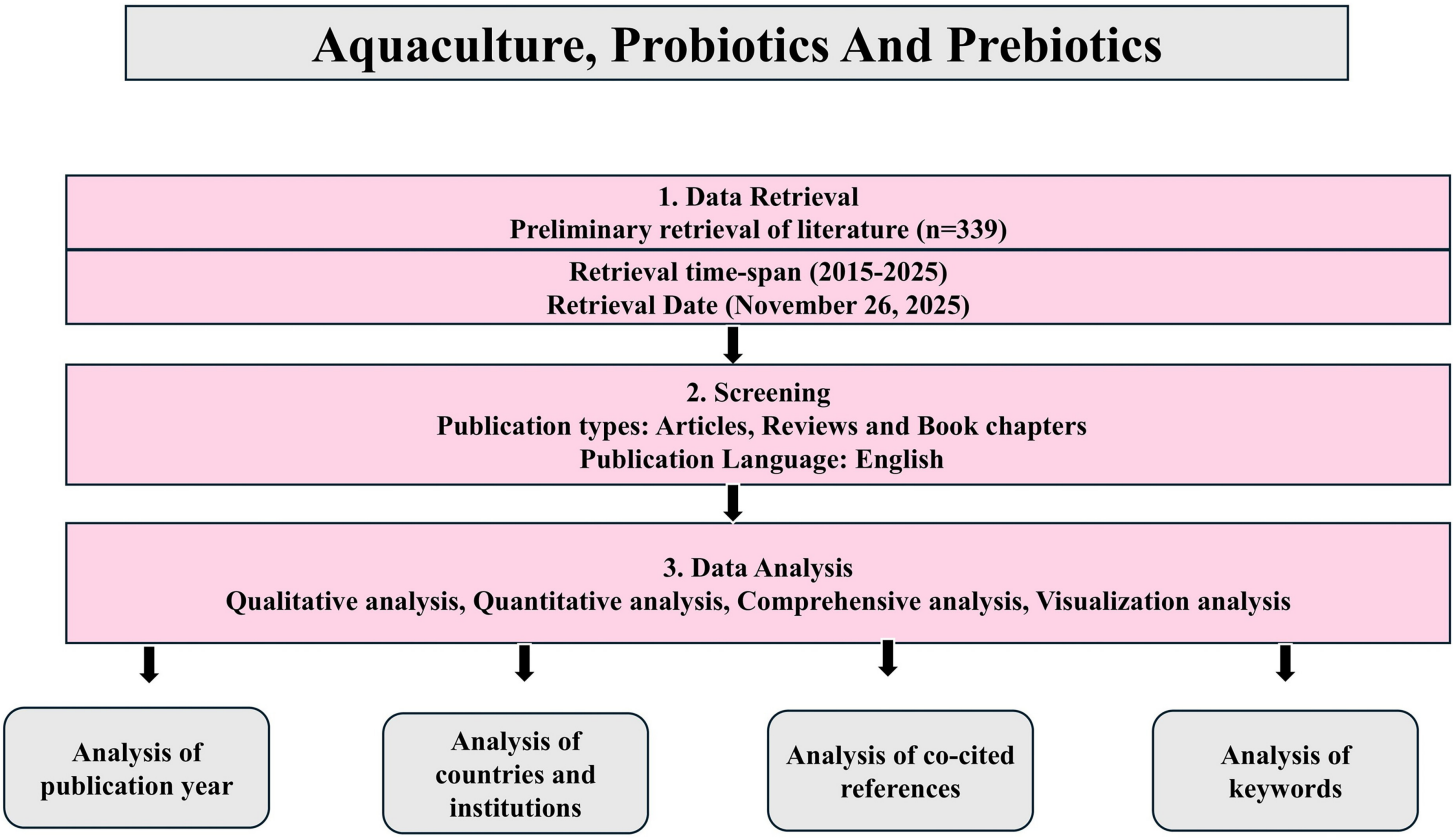
PRISMA flow diagram on data screening and inclusion.

A detailed review was conducted by examining the literature on the Scopus database. Scopus was selected because of its reputed profile and widely used database of literature in review and bibliometric analysis. The search for the literature on aquaculture probiotics and prebiotics was retrieved from Scopus database using the subject (TITLE-ABS-KEY (aquaculture probiotic prebiotics) AND PUBYEAR > 2004 AND PUBYEAR < 2026 AND PUBYEAR > 2004 AND PUBYEAR < 2026 AND (LIMIT-TO (DOCTYPE, “ar”) OR LIMIT-TO (DOCTYPE, “re”) OR LIMIT-TO (DOCTYPE, “ch”))). Figure 1 shows the data retrieval and collection from the search engine Scopus. VOS Viewer (version 1.6.20, copyright at 2009–2023) was a functional and efficacious tool used for analyzing the bibliometric analysis. The network visualization of “co-occurring keywords” plays a pivotal role as it is an important for the research area. The more the recurrence of the keywords the more it reveals the more research carried out in that research field. The software constructs maps were based on the related data. The network build will analyze the title, keywords and word recurrence quantitatively.

Bibliometric analysis is a notable and systematic tool used for thorough examination and interpretation of large amounts of data. This analysis helps the researcher to analyze the large amounts of publications, including various institutions, countries and research areas. Data was collected from Scopus covering the publications from 2005 to 2025. This was done by screening Scopus based on titles, abstracts, and keywords. The metadata comprised 339 research articles, book chapters and reviews that contained one of the keywords searched. The bibliometric analysis includes names, countries, keywords, publication dates and were exported. VOS viewer was further used to analyze and visualize the keyword clustering networks.

## Results

**FIGURE 2:**
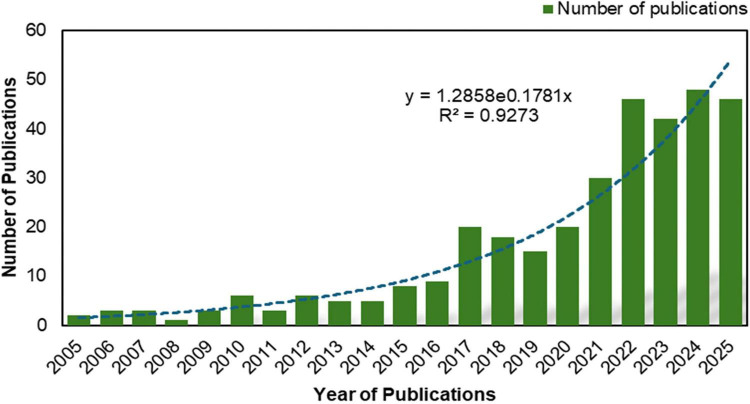
Publication progress over the year (2005–2025) based on the SCOPUS documents (aquaculture, probiotics, prebiotics).

A total of 339 publications and book chapters were collected from Scopus where the top five subject categories were agricultural and biological sciences, immunology and microbiology, environmental science, biochemistry, genetics and molecular biology and medicine had authored to 69.6, 25.3, 24.4, 20.6, 12.9% of all metadata publications (339), respectively. Figure 2 shows the global publication trends between the publication numbers and year. From the year 2017, a remarkable increase in the number of publications was observed. The year 2024 was found to have ranked the highest number of publications with 48 papers, followed by the years 2022 and 2025 with 46 papers each.

### Analysis of countries and regions

**FIGURE 3:**
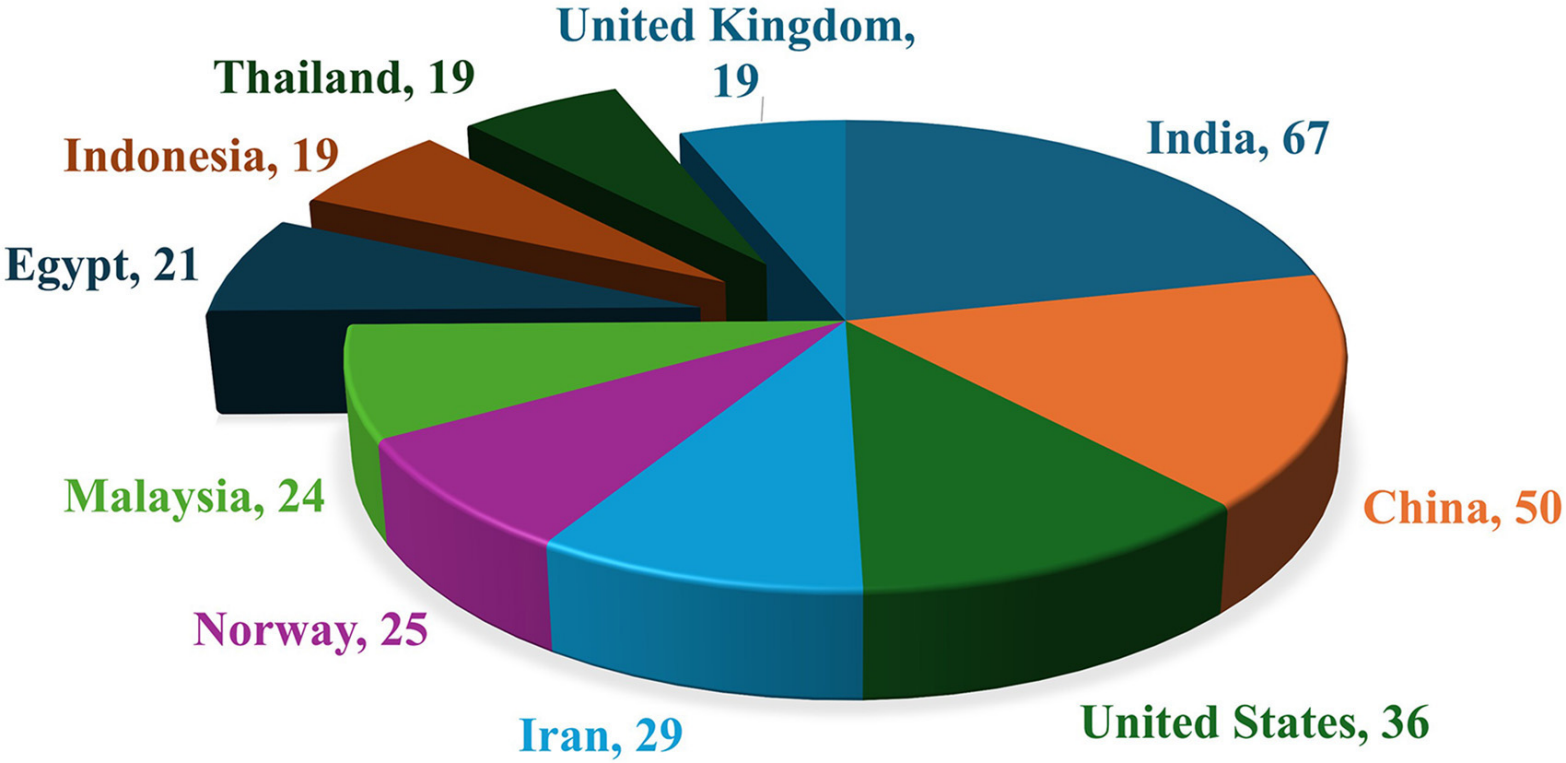
Publications on aquaculture, probiotic, and prebiotics from different countries (2005–2025).

Totally 72 countries have published at least one research article on keywords searched based on Scopus database. The maximum number of articles were from India with 67, followed by China and United States with 50 and 36, respectively. Figure 3 explains the highly published research articles from different countries.

### Vos viewer analysis

**FIGURE 4:**
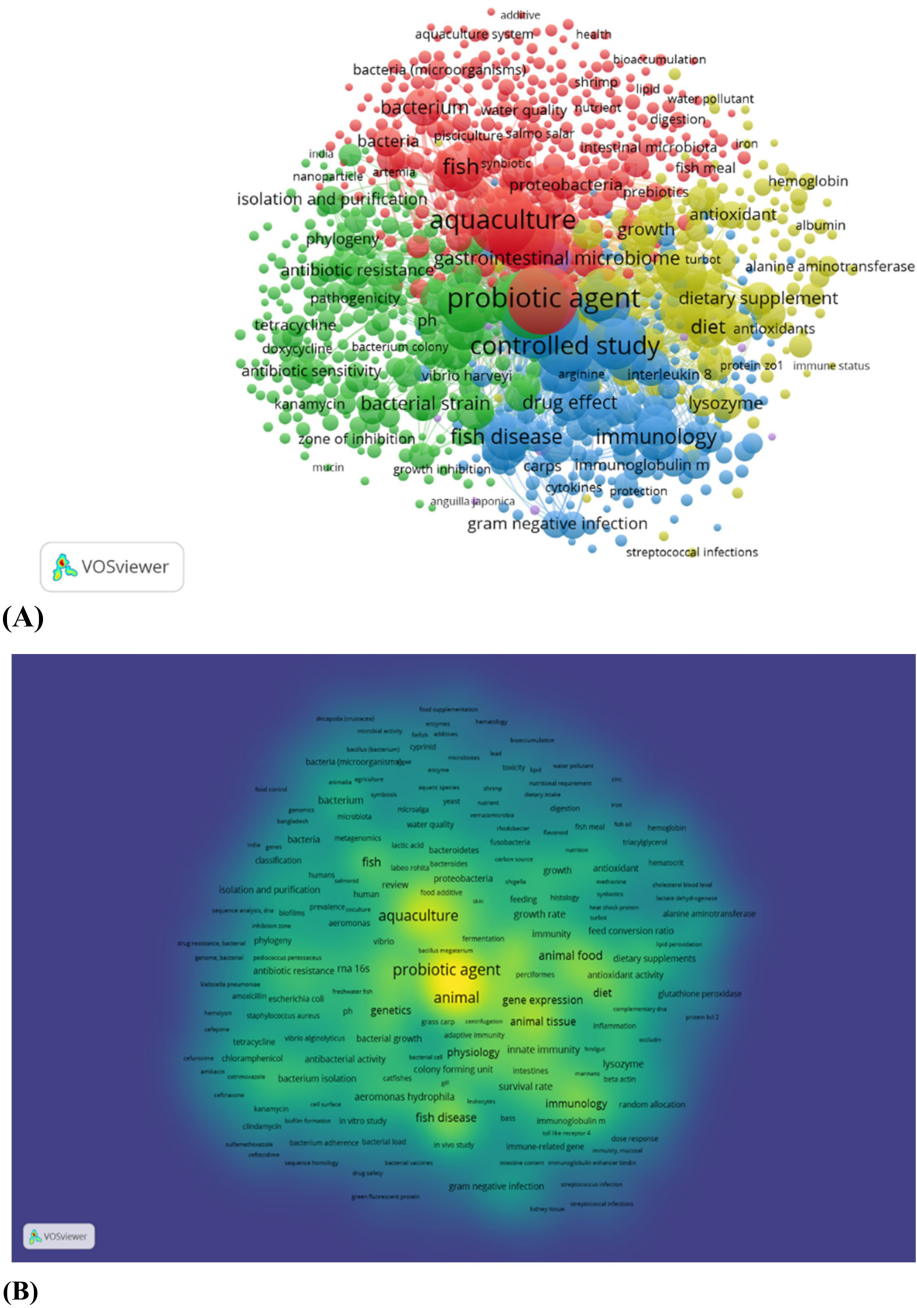
**(A,B)** Co-occurrence network visualization and density view of the SCOPUS based databases using VOS viewer software for “aquaculture” and “probiotic.”

A total of 1,475 articles, reviews and book chapters were collected from the Scopus database from the period 2015 to 2025 for the visualization of co-occurrence of the keywords. The data are drawn in CSV format and then loaded to VOS Viewer for the network visualization. The analysis was set to “co-occurring” and all keywords. The threshold value was five, the number of times will reoccur. The visualization frequency of “aquaculture” and “probiotics and “fish” were observed. The major keywords were “probiotic agents,” “immunology,” “antimicrobial activity,” “dietary supplementation,” fish diseases, water quality.” The size of each word represents the more recurrence of the keywords, and the color represents the cluster which they belong. The thickness of the lines represents the maximum number of interactions between each of them. There is a total of four clusters observed in the Figure 4A, which explains the major articles. Cluster 1 (red) explains probiotic study and microbial community and the effect on aquaculture system. Cluster 2 (green) covers probiotic effect as an antibacterial agent and how it can substitute antibiotics used in aquaculture. Cluster 3 (blue) covers the probiotics treatment in aquaculture enhances the immune responses in host and immune-related genes. Cluster 4 (yellow) shows the dietary supplementation in aquaculture sector. Cluster 5 (purple) is the least related cluster showing antibiotic therapy and gastrointestinal microbiome. The density view of the co-occurrence network observed in the Figure 4B. According to cluster analysis, a number of studies have been conducted related to probiotic isolation and treatment in aquaculture and its efficacy. A future scope is on dietary supplementation with probiotics and its effect on the gastrointestinal microbiome.

## Discussion and future perspective

### Probiotic

The term “probiotic” is a Greek word which means “prolife” or “for life” coined by Lilly and Stillwell (1965). In 1905, Metchnikoff (1907) was the first to describe the constructive role played by some bacteria among farmers who consumed pathogen-containing milk and that reliance on gut microbes for food makes it possible to take steps to change the flora of our bodies and to replace harmful microbes by beneficial microbes. However, the term probiotic was introduced only by Lilly and Stillwell (1965) as a modification of the original word “probiotika.” The definitions about probiotics emphasize on the point that the probiotics are alive and preferably active. However, in recent years, findings show that non-viable, inactivated, and even ruptured cells of probiotics have therapeutic effects on the host metabolism. However, safety related complication of live microbial cells is in concern. In recent studies the new terminology was introduced, and probiotics are redefined as true probiotics, ghost probiotics and pseudoprobiotics based on their viability and functionality (Zendeboodi et al., 2020). The byproducts of immune system like DNA, cell wall components and metabolites are influenced by probiotics. The regulation of immune system can be affected by non-living probiotics and their release (Oelschlaeger, 2010). Virus, parasitic and bacteria are the main causes of diseases in aquaculture (Carbone and Faggio, 2016; Bastos Gomes et al., 2017). Studies revealed that most of the pathogenic diseases in aquaculture are often associated with the genus *Aeromonas, Vibrio, Yersinia, Streptococcus, Acinetobacter, Pseudomonas, Lactococcus, and Clostridium* (Santos and Ramos, 2018; Yi et al., 2018). The growth rate of aquatic animals has been improved through several strategies like feed, health optimization and environmental improvements. Probiotics played an important role as growth promoters in this. Nutrient competition is one of the methods in which probiotic microorganisms compete with aquatic pathogens for the essential growth nutrients. Vibriosis is caused by *vibrio* species, a common epizootic disease that affects wild and farmed fishes worldwide (Gao et al., 2017). The strain *Pedicoccus acidilactici* was isolated from the gut of *Oncorhynchus mykiss* (Araújo et al., 2016). In this study, strains were showing antibacterial activity against common fish pathogens due to the production of bacteriocin. Antibiotic resistance was not observed in this strain instead of hemolysins are produced or degraded gastric mucin (Araújo et al., 2016). Probiotics alleviate lactose intolerance, with their potential to regulate the immune system and prevent various chronic disorders. The major consequence of probiotic supplementation is the production of digestive enzymes such as protease, lipase, amylase, and lysozyme which improve nutrient absorption and gut microbiota (Yousefi et al., 2019). Improved digestibility of certain nutrients lowers blood lipid concentration and reduces food intolerance (Ringø et al., 2018). Recently, scientists have formulated machine learning models which analyze genetic data and tRNA sequences that differentiate beneficial bacteria and intestinal pathogens which help in targeted probiotic treatments (Bergamini et al., 2022). Biotechnology and information science, Artificial intelligence and Machine learning are now being widely used for better understanding of intestinal microbiome (Han et al., 2025).

#### Impacts of probiotics on gut microbiome

Adherence of Probiotic microorganisms to the intestinal epithelial lining of the host is a major part of the probiotics selection that can increase their survival in the gastrointestinal tract with beneficial effects (Mirzabekyan et al., 2023).

The multifaceted community of gut microbiota has many microbes in the human colon. Their metabolism regulates the host homeostasis (Chandrasekaran et al., 2024). The gut microbiome and humans have a co-inhabitant behavior. The major strains of bacteria in human gut microbiomes include *Proteobacteria, Bacteroidetes, Verrucomicrobia and Firmicutes* (Rinninella et al., 2019). The microbial diversity in gastrointestinal tract is different, as human colons have the highest populations and only a few species are present in the human intestine and stomach (Thursby and Juge, 2017). External triggers such as diet and antibody usage can influence the gut microbiome (Hollins and Hodgson, 2019). Short Chain Fatty Acid (SCFAs), amino acids, trimethylamine N-oxide, bile acids, tryptophan and indole derivatives are major microbiome derived compounds (Agus et al., 2021). Metabolomics is a study which is used to analyze the small molecules in the body fluids. People with metabolic disorders are due to the changes in their metabolites. These changes often correlated to disturbances in the gut microbiota (Koh et al., 2018). Because of tight junction proteins and increased mucin production the *Lactobacillus* species could enhance gut barrier integrity (Dempsey and Corr, 2022). The inflammatory bowel diseases (IBD) are regulated by regulatory T-cells (Treg) and cytokines such as interleukin-10 (IL-10) which act as immunomodulators (Su et al., 2024). Bifidobacterium species help in the digestion of dietary fibers that the body cannot digest. During this fermentation process SCFAs are produced which can nourish the colonocytes, maintain gut lining and reduce inflammations in the gut (Yoon et al., 2023). Next generation probiotics are now emerging as they are more susceptible to the unfavorable conditions in gastrointestinal tracts (Jan et al., 2024). Traditional probiotics do not target diseases such as colitis, obesity, liver disease and diabetes (Jan et al., 2024).

#### Soil probiotics

These are the microorganisms which originate from the soil and enhance soil fertility. Recent research on soil-based probiotics focusses on bacteria like *Bifidobacterium* and *Lactobacillus*, and their role in soil ecosystem. In agriculture sectors, soil-based probiotics have enhanced plant growth, soil fertility, and sustainable farming practices (Wang et al., 2022). The study by de Oliveira et al. (2024) reported that the commercial application of probiotic in shrimp pond has bioremediation property and enhances the soil microbiome. The identified soil probiotics and its sources are given in Table 1. Soil microbiota function as a natural regulator of Earths carbon homeostasis. Well resilient agricultural practices can elevate the soil microbiota and sequester carbon which is formed from the microbial breakdown of complex organic molecules (García et al., 2025). However, the traditional agricultural practices such as deep tillage and monocropping can disturb microbial activity which in turn results in the release of carbon into the atmosphere (Thotakuri et al., 2024; Babaniyi et al., 2024). Climate change also has a pivotal role in the change in soil microbiomes affecting their nutrient cycle, carbon sequestration, and crop productivity (Bardgett and van der Putten, 2014; Pugnaire et al., 2019). Using bio stimulants in the agricultural sector is an environmentally friendly strategy that enhances the soil fertility (Bargaz et al., 2018). These bio stimulants contain potential probiotics that increase the productivity (Gavelienė and Jurkonienė, 2022). *Bacillus* species are used to treat cereals which improved root growth and plant growth resulting in increases in crop yield and soil fertility (Dal Cortivo et al., 2020).

**TABLE 1:** List of identified soil-based probiotic (SBPs).

No.	Soil-based probiotic	Description	References
1	*Bacillus coagulans*	Produces lactic acid- microbial metabolite that supports gut health	Majeed et al., 2015; Jäger et al., 2018; Madempudi et al., 2020
2	*Bacillus subtilis*	It is known as grass *bacillus* or hay *bacillus* because it is found in the gastrointestinal tract of cows	Hanifi et al., 2015; Lefevre et al., 2015; Townsend et al., 2018
3	*Bacillus clausii*	Reduce nitrate in the soil to nitrite	Lippolis et al., 2013
4	*Bacillus indicus*	These bacteria can produce a characteristic yellow-orange pigmentation	–
5	*Bacillus licheniformis*	Mostly found in the feathers of birds	–
6	*Enterococcus faecium*	Commensal forms in human and animal gut causes diseases like neonatal meningitis or endocarditis	Yermolenko et al., 2006; Surono et al., 2011
7	*Clostridium butyricum*	Predominantly used probiotic in Asian countries	Kato et al., 2018

#### Role of probiotics in aquaculture

Probiotics are isolated from aquatic animals where water and other biotic agents act as the carriers for their spreading and once they enter the host body these microorganisms perform critical physiological functions. Studies have shown that both in humans and animals, microbiota plays an essential role in proper development and defense against pathogens (Banarouei et al., 2019). The probiotic in aquaculture interprets dual benefits- on host microbiota and the aquatic environment, thereby optimizing feed utilization, fish health and growth rates (Verschuere et al., 2000). Studies demonstrated that the use of probiotics in their functional feed can improve the health of aquatic animals, devoid of negative side effects (Pérez-Ramos et al., 2018). The most common species of probiotics used in aquaculture include specific strains of yeasts and bacteria. There are many beneficial bacteria which produce enzymes such as amylase, by *Bacillus subtilis, Aeromonas spp*., *Clostridium spp., Bacteridaceae, Staphylococcus sp.*, and *Lactobacillus plantarum*, and proteases and cellulose by *B. subtilis, Staphylococus sp*., and *L. plantarum* (Chantharas et al., 2011). According to Verschuere et al. (2000) farmed fish and shellfish are susceptible to diseases caused by microorganisms present in the surrounding water bodies as they are close in contact with the water surface and ingest it continuously. Opportunistic pathogens like *Vibrio spp.* invade into the fish gut through their body organs like gills and skin (Weber et al., 2010). During the last decade, several studies on probiotics have been published. As per the literature review, the probiotic species such as *Pedicoccus, Saccharomyces cerevisiae, L. casei, Clostridium butyricum, L. planta, B. subtilis, and Holobacterium salinarum* showed substantial improvement in aquaculture species through many aspects like improving their immune responses, growth and feed utilization (Mohammed et al., 2025a). Probiotics secrete different antimicrobial substances like bacteriocins, hydrogen peroxide, bacitracin and siderophores which reduced the activity and growth of pathogenic bacteria (Yousuf et al., 2023). Certain bacteria can thrive in iron-deficient conditions efficiently by producing siderophores (Kesarcodi-Watson et al., 2008). Previous studies have reported that *Bacillus* species synthesize bacteriocins and siderophores that have suppressive ability against fish pathogens. *B. thuringiensis* strain BMG1.7 produces novel bacteriocin, named thuricin 7, which acts as an antimicrobial agent (Cherif et al., 2001). Use of probiotics in aquaculture helps in the reduction of harmful algal growth concentration of organic matter concentration, and increased oxygen levels in water. Fish were supplemented with probiotics and analysis of histological parameters of intestine showed a thick mucosal layer and increased number of goblet cells which shows enhanced gut health of fish (Feng et al., 2025). Probiotics can increase the production of mucus from goblet cells in the intestinal lining which can act as a protective outer layer for the invasive pathogens (Ng’onga et al., 2025). Fish guts are a key organ in digestion, nutrient absorption, and immune defense as it is a highly flexible and dynamic organ system (Ntakirutimana et al., 2023). Use of probiotics increases the number of villi which helps in the absorption of nutrients by creating a morphological modification (De Marco et al., 2023). The probiotic interaction is mostly with the host immune system, gut epithelium and gut microbiome, resulting in the alteration of gut histology (Auclert et al., 2024).

#### Antimicrobial activity

The antioxidant and antibacterial activity of the methanolic extracts of freeze-dried cells of probiotic *Lactobacillus* strains were investigated using molecular analytical techniques (Richards et al., 2020). It was reported that the highest antibacterial activity was seen in the crude extract of *Lb. rhamnosus* strains and *Lb. casei* strain. The current focus research focuses on the discovery of next-generation probiotic strains or novel strains exhibiting antimicrobial properties that can efficiently modulate the ecological taxa composition and functionality of the human microbiota in the gut and its further aspects (Fijan, 2023). Ahmed and co-authors investigated that certain *Weissella* species exhibit antimicrobial and anti-inflammatory effects that are found to be clinically treatable bacteria with emerging antimicrobial and probiotic associated health benefits (Ahmed et al., 2022). Several lactobacilli were isolated from different sources and gene pools with bioactive secondary metabolites were identified. The secondary metabolites like terpenes, non-ribosomal peptide-synthetase, lanthipeptide, LAP, T3PKS were identified the which help the probiotic to survive under different stress conditions (Mukesh Kumar and Dhanasekaran, 2021).

#### Role of antioxidant activity on the probiotic bacteria

Probiotic bacteria have prominent antioxidant activity which includes active antioxidant signaling pathway, stimulation of antioxidant enzymes, ROS binding systems, metabolite synthesis, intestinal barrier integrity, induction of oxidative damage repair system and sustained mitochondrial activity (Xiao et al., 2025). Reactive oxygen species (ROS) are the oxygen-containing free radicals and peroxides related to living organisms in the form of hydroperoxide, superoxide, hydroxyl radical and singlet oxygen (Averill-Bates, 2024). Many studies have reported that the probiotic variants of bacterial strains have different antioxidant capacity. In the study of Kullisaar et al. (2002) it was found that lactobacillus fermentum E-3 and E-18 were able to express Mn-SOD to resist oxidative stress. A study conducted by Castex et al. (2009) reported that *L*. *stylirostris* fed a diet enriched with 1 g/kg^–1^ of *Pedicoccus acidilactici* MA18/5M for 1 month and reported an increase in survival rate, lower oxidative stress in their digestive gland and changes in total antioxidant status (TAS). In another study of Castex also reported that after natural infection by *Vibrio nigripulchiritudo*, probiotic-fed shrimps showed an increased survival rate and lower OS damages in their digestive gland. It was also reported that ROS production is a part of the immune defense system and plays an important role in microbicidal activity (Zambón et al., 2000). Xiao et al. (2025) reported that *L. rhamnosus* KKU-D89 showed high probiotic potential in different assays including acid and bile salt tolerance, hydrophobicity, antimicrobial activity and antibiotic susceptibility. LABs have prominent role in oxidative stress-related disease models and metabolic disorder (Xiao et al., 2025).

### Prebiotics

Non-digestible food ingredients that can beneficially affect the host by selective stimulation of the growth and/or the activity of one or a limited number of bacteria are called prebiotics. Research on the use of prebiotics in fish farming and shellfish have received limited attention. Prebiotics are carbohydrates which are classified based on their molecular size based on their degree of polymerization in number of monomeric units of monosaccharide units. According to the International Union of Pure and Applied Chemistry nomenclature, oligosaccharides are defined as saccharides containing between 3 and 10 sugar moieties (Mussatto and Mancilha, 2007). In the review of Akhter et al. (2015) it was reported that prebiotics act as a growth factor to specific commensal bacteria, that inhibit the adhesion and invasion of pathogenic microorganisms in the epithelium lining of colon by competing for the same glycol-conjugates found on the surface of epithelial cells, by inducing cytokine production, preference of short chain fatty acids and altering the pH of the colon. Prebiotics directly enhances the innate immunity. However, prebiotics which are incorporated as feed additives have a capability to overcome antibiotics misuse in aquaculture species health. Feed additives have been widely reported to give promising results in improving growth (Ramos et al., 2017), enhancing immune response (Selim and Reda, 2015), disease resistance (Li et al., 2022) and relieving abiotic stress of aquaculture species (Hoseinifar et al., 2014). In human gut microbiota, prebiotics have a selective and indirect impact. Recent studies have reported that the nutrient source of prebiotics exhibits a regulatory effect on the beneficial gut microbiota (Ayad et al., 2025). Many studies show that prebiotics have an emerging effect on the B-cell responses (Rousseaux et al., 2023). Gut microbiota can degrade the dietary carbohydrates that human digestive enzymes cannot break into digestible nutrients. This is known as “an energy harvesting” system (Bedu-Ferrari et al., 2022). The prebiotics can cause changes in gut microbiota which can affect different microbes in the microbial community which is due to the cross-feeding interactions and functional redundancy (Bindels et al., 2015).

#### Types of prebiotics

A wide range of prebiotics are identified. Most of them are forms of carbohydrate groups and belong to the class oligosaccharides. There exist different natural dietary food products which include asparagus, garlic, sugar beet, honey, banana, onion, Jerusalem artichoke, wheat, barley, tomato, rye, humans and cow milk, peas, beans etc. and recently seaweed and microalgae which are reported to be producers of prebiotics (Varzakas et al., 2018). The origin of prebiotics can be either from plants or animals. They can be extracted or produced from microbial sources and are synthesized chemically and enzymatically.

#### Naturally occurring prebiotics

Prebiotics are naturally occurring biopolymers which are formed with enzymatic activity which helps with various functions like production of biofilm, protection from environmental stress and immune response of host species (Marathe, 2025). Arabinoxylan oligosaccharide was produced from wheat bran with the use of *Bacillus subtilis* (Swennen et al., 2006). The growth of probiotic *Bifidobacterium* species is promoted by the hemicellulose substance from plant cell wall galactoglucomannans. This is obtained from wood-based plants (Polari et al., 2012). The most studied fructo-oligosaccharides (FOSs) and GOSs could enhance the growth of intestinal bacteria like *Bifidobacterium* and *Lactobacillus* (Roberfroid et al., 2010; Gibson et al., 2017; Tandon et al., 2019).

#### Fruit waste

In aqua-feed, added-value and bioactive components are produced from fruit processing by-products. Studies by Veneziani et al. (2017) reported that the source of bioactive compound can be any part of plants. Processed fruit by-products contain bioactive compounds which have many biological properties that are beneficial for aquaculture. Fruit by-products contain many bioactive nutrients like flavonoids, polyphenols, anthocyanin, pigments, tannin, glucosinolates, dietary fiber and saponins (Leyva-López et al., 2020). In the review of Leyva-López et al. (2017) the bioactive derivatives of fruit by-products and their anti-oxidative and immuno-stimulant functions in aquatic animals are discussed. Apple juice is fermented to produce apple cider with yeasts as it is the fermentation of sugar into alcohol (ethanol) and with the activity of *Lactobacillus* (Călugăr et al., 2023). Malolactic fermentation (MLF) is mostly with LAB. Therefore, in MLF the L-lactic acid formed from the L-malic acid which increases the cider’s sensory characters which reduces the acidity and increases the microbial stability (Kristof et al., 2023). The processed products from fruit mainly apple and peach fruit pomaces can be used as a functional active substance of yogurt (Demirkol and Tarakçi, 2025). It has been reported that citrus peels have more abundant functional ingredients like fiber, oligosaccharides, and antioxidants (Lario et al., 2004). In recent studies, it was shown that *Passiflora edulis* is composed of secondary metabolites like polyphenols, flavonoids and anthocyanins which are responsible for its antibacterial and antioxidant activities (Viera et al., 2022).

#### Seaweed

Seaweeds are rich sources of bioactive compounds which include many novel dietary fibers, carotenoids, fatty acids and polyphenols. It has been reported that a substrate that is selectively utilized by host microorganism conferring a health benefit (Gibson et al., 2017). The total amount of polysaccharide in seaweed varies from 2.97% to 71.5% (Cherry et al., 2019; De Jesus Raposo et al., 2016). The seaweed protein includes lectins, glycoproteins, and phycobiliproteins (Echave et al., 2022). A study reported that seaweed and functional metabolite supplements derived from seaweed have a positive effect on physiological stress resilience, growth, and immune response in fishes (Siddik et al., 2023). The commercial seaweed liquid extract True-Algae-Max (TAM), from *Ulva lactuca*, *Jania rubens*, and *Pterocladia capillacea* was given as dietary supplement to *O. niloticus* which showed enhanced growth performance and immune responses when challenged with *A. hydrophila* (Ashour et al., 2020). The strains on wild fish populations can be lessened by reducing reliance on fish-based ingredients in fish feed by adding seaweed-derived nutraceuticals. Research and development in this field has reported that seaweed-based remedies have a lot of potential to help aquaculture become more resilient and sustainable in future. Recent studies have shown that fermented seaweed improves the fish’s health and its performance (Kurian and Paari, 2025). Fermentation study of the seaweed showed resistance against pathogenic bacteria *Vibrio harveyi* and *Aeromonas hydrophila*. Oxidative stress plays a critical role in the disease resistance of fish (Peng et al., 2022). However, an effective functional feed enhances infection resilience against pathogenic challenges. The bacteria that are associated with seaweed are beneficial for both seaweed and bacteria. These bacteria produce some bioactive substances that help the growth of seaweed, cell density dependent signaling and many other molecules (Oliveira et al., 2014; Stevanović et al., 2018). The antibacterial compounds produced by these seaweeds provide self-defense mechanisms and can also be an alternative to the antibiotics in the pharmaceutical industry (Setyati et al., 2025).

#### Herbal extracts

Natural products are pivotal for optimum health, anti-inflammatory properties and antioxidants since they contain bioactive metabolites like flavonoids, alkaloids, phenolics, steroids, volatile oils and vitamins, minerals and fiber, and vital nutrients (Aly et al., 2023; Cusumano et al., 2024; Goher et al., 2024; Zengin et al., 2024). Studies showed that bacterial diseases affect animals, which affect livestock economically. However, antibiotic growth promoters (AGPs) are a causative factor in the resistance to bacterial pathogens (McGaw, 2025). Therefore, one line of action could be the use of plant extracts or essential oils which provide beneficial effects like antibacterial activity and stimulate immune response etc. The bioactive compounds which are present in aromatic herbs can affect biological systems. These bioactive compounds are fixed and volatile secondary metabolites (Grigore-Gurgu et al., 2025). Aromatic herbs are widely used for their antibacterial and antioxidant properties (Grigore-Gurgu et al., 2025). Phytochemical composition and phytochemical properties are influenced by environmental factors and genetic factors (Martins et al., 2015). The Green solvent and organic solvent extraction give bioactive phytochemicals (Kumar et al., 2023). Herbal extracts are mainly used in pharmaceuticals and textiles. In the food industry, many herbs like rosemary, thyme, marjoram and oregano are used because of their antimicrobial and antioxidant properties. In the study of synergistic concentration of oregano and rosemary essential oil emulsions in the Minas cheese showed change in the growth of *E coli* with zero effect on *Lactobacillus acidophilus* (Diniz-Silva et al., 2020). Encapsulated bioactive compounds from the extracted herbs are used as feed additives, reducing oxidative stress and enhancing the target delivery to gut of the animals (Stevanović et al., 2018). As these herbal extracts or oils are less toxic and safe to use and they can be used as substitute to chemical pesticides (Stevanović et al., 2018). Application of nanotechnology to produce biopesticides has high efficiency and has a significant effect on human-animal health and the environment (Oliveira et al., 2014).

## Conclusion

Probiotics have been studied and explored in different forms as a commercial product. Many recent studies demonstrate that probiotics and prebiotics have beneficial effects in humans and animals. There exist many non-pathogenic organisms that are used as probiotics which can adhere, colonize and modulate intestinal property. Prebiotics can enhance the growth and activities of probiotics. However, the combination of probiotics with prebiotics incorporated as feed can have a high impact on infectious diseases in aquaculture. The synergistic effects of probiotics and prebiotics have a prominent role in commercial aquaculture and can increase the total health of aquatic animals. Nonetheless, proper dosage and types of prebiotics must be taken into consideration and clinical trials should be conducted.

## Data Availability

The original contributions presented in this study are included in this article/supplementary materials, further inquiries can be directed to the corresponding author.
